# Establishment of a stable proficiency testing matrix in transfusion microbiology in South Africa

**DOI:** 10.4102/ajlm.v12i1.2095

**Published:** 2023-08-30

**Authors:** Xoliswa L. Mpumlwana, Winnie Kruger, Ute Jentsch

**Affiliations:** 1 Department of Quality Control, South African National Blood Service, Roodepoort, South Africa; 2 Department of Biomedical Sciences, Faculty of Health Sciences, University of Johannesburg, Johannesburg, South Africa; 3 Department of Specialized Lab Services and Quality Control, South African National Blood Service, Roodepoort, South Africa

**Keywords:** South African National Blood Service, bacterial contamination, transfusion-transmissible bacteria, proficiency testing matrix, bacterial stability testing

## Abstract

**Background:**

All medical laboratories must participate in proficiency testing (PT) programmes to ensure high-quality results. Proficiency testing samples mimic clinical samples; however, PT programmes for detection of bacteria in blood products are not routinely performed due to unavailability of matrix-equivalent samples.

**Objective:**

The aim of this study was to develop and test a matrix-equivalent PT programme using blood products as the basis matrix.

**Methods:**

A prospective cross-sectional study was conducted from April 2021 until June 2021, using 52 blood products comprising 36 pooled platelet and 16 red blood cell products at the South African National Blood Service PT laboratory in Gauteng. Products were manipulated into matrix-equivalent PT samples by spiking 42 products with known bacterial strains at specific concentrations and treating the remaining 10 products with preserving fluid containing antibiotics. The level of agreement between the researcher results and participating laboratories’ results was assessed.

**Results:**

Of the prepared matrices, 568 out of 572 (99%) were stable for 30 days. Bacteria could correctly be identified in spiked samples for up to 23 days. Samples treated with preserving fluid remained negative until day 30. For spiked samples, an average of 98% agreement (153/156) was achieved between the three participating laboratories when compared with the researcher’s results; 100% agreement was achieved for unspiked samples. The kappa scores obtained from all tested variables presented with scores between 0.856 and 1.000, and the *p*-value was < 0.001 throughout.

**Conclusion:**

The developed PT matrix was therefore stable and suitable to be implemented in transfusion microbiology.

**What this study adds:**

This study demonstrated that a stable microbiology PT programme using platelets and red blood cells can be developed for use on bacterial detection analysers and could help to close the gap presented by unavailability of a blood PT matrix for transfusion microbiology.

## Introduction

Blood products provide therapeutic and life-saving benefits to the recipients. International best practice guidelines provide evidence-based information on how to mitigate against transfusion-transmitted bacterial infections and assure blood safety from collection to issuing. Despite these measures, which include screening of donors prior to blood donation, use of effective antiseptics, screening of donated blood for viral markers and aseptic technique during processing of blood and blood products, bacterial contamination of blood products, especially of platelets, remains a significant infectious hazard in blood transfusion.^1,2,3^ While viral causes of transfusion-transmissible infections are effectively screened for, bacterial contamination of blood products remains a significant risk. Platelets, when compared to other blood products, are more prone to bacterial contamination because of their storage conditions at 20 °C – 25 °C, which are conducive to bacterial growth.^3,4^ Quality control testing of platelets and other blood products in microbiology using bacterial culture and bacterial identification methodologies have been implemented in the blood transfusion field as a further mitigating practice.^5^ These screening methodologies have shown effectiveness and have decreased events of transfusion-transmitted bacterial infections due to early detection of bacteria in contaminated products.^6,7^ Rapid classification and detection of microbes is essential for the prevention of treatment failures and rapid spread of antimicrobial drug resistance.^8^

The South African National Blood Service (SANBS) quality control testing for bacterial screening is performed on 1% of the donated apheresis and pooled platelet (PP) products.^9^ The SANBS and other blood transfusion services are experiencing a quality gap as there are no formal microbiology proficiency testing (PT) matrix programmes for blood product safety testing. Lyophilised and frozen commercial PT programmes were validated for use in verification of the sensitivity of bacterial detection instruments.^10^ These commercial PT programmes do not fully cover the matrix effect as some human manipulation is still required during the testing process; they require either thawing or making up volume with sterile water. The PT matrix is the totality of components within the sample in addition to what is being measured and represents real-time samples in terms of viscosity, turbidity, composition and colour.^11^

This study developed and tested a blood product-based PT matrix as a foundation to develop a full programme in the future. The developed matrix did not require any manipulation by the participating laboratories; all matrix component requirements were met. The requirements of this matrix are that it must be stable and support bacterial growth.

## Methods

### Ethical considerations

This study was approved by the Faculty of Health Sciences, University of Johannesburg (HDC-01-3-2019), and the SANBS Human Research Ethics Committee (HREC, SANBS: 2019/0476). Donors provided consent prior to the blood donation process for using some of their blood for blood safety improvement purposes. The participating laboratories were invited and gave consent to take part in the study as independent evaluators of samples. Anonymity and confidentiality were maintained by giving each donation a unique identifier and data were collated in a Microsoft Excel (Microsoft Corp., Redmond, Washington, United States) spreadsheet, decoded, and stored electronically in a password-controlled folder.

### Sample population

The study took place in the SANBS proficiency testing laboratory in Johannesburg, South Africa, between April 2021 and June 2021. Whole blood units donated by eligible blood donors were collected by qualified phlebotomists using SANBS-approved protocols. Donated whole blood units were couriered at temperatures between 1 °C and 10 °C by the SANBS transport department from the donor centres to a centralised SANBS processing site. Each whole blood unit was processed and separated into red blood cells (RBC), platelets and plasma components. Thereafter, five platelet units from five different whole blood donors with the same blood group were pooled together to make one PP unit. The statistical value for sample size was calculated based on a method used on two-rater studies to detect a statistically significant kappa on dichotomous variables.

Based on the availability at the time of sampling, blood group A+ units were mostly used in this study as there were excess units available and using these would not have a negative impact on blood supply.^12^ Pooled platelet units (*n* = 36) and excess A+ RBC packed-cell units (*n* = 16) were produced for the purposes of this study and sent to the PT laboratory for the production of blood matrix PT samples.

### Study design, sample testing, materials and instruments

This was a prospective cross-sectional study that was approached in two phases, namely the preparation of the PT matrix and the testing of the prepared PT matrices.

### Preparation of proficiency testing matrices

Blood product units consisting of PP and RBC concentrates were obtained and tested for baseline sterility prior to manipulation into PT matrices. No additional supplements were added to the concentrates while conducting the study. After sampling for baseline sterility testing, 42 of the 52 units, comprising 29 PP and 13 RBC concentrates, were each spiked with 1 mL of a known bacterial strain using coupled spikes. The selected bacterial strains known to cause transfusion-transmitted bacterial infections included *Staphylococcus epidermidis, Staphylococcus aureus, Streptococcus pyogenes, Streptococus bovis, Streptococcus dysgalacticae, Bacillus cereus, Bacillus thuringiensis, Listeria monocytogenes, Enterobacter cloacae, Serratia marcescens, Escherichia coli, Morganella morganii, Pseudomonas fluorescens, Klebsiella pneumoniae* and *Serratia liquefaciens.*^13^ The bacterial concentrations ranged between 0.5 and 0.63 MacFarland standard, utilising a variety of common Gram-positive and Gram-negative bacteria. Only one bacterial strain was introduced into each product unit or concentrate during inoculation. The bacterial strains used were validated by the Paul Ehrlich Institute and are available at the World Health Organization as reference strains for use in blood transfusion services.^14^

The remaining unspiked 10 units were treated with 1 mL preserving fluid containing bacteriostatic agents. The use of the preserving fluid was to ensure that the non-spiked units remain negative until 7 days after the closing date.^13^ The PP units were stored at 20 °C – 25 °C while RBC units were stored at 2 °C – 6 °C for 48 h, which are the standard storage conditions for these products to allow bacterial proliferation to take place.

**FIGURE 1:**
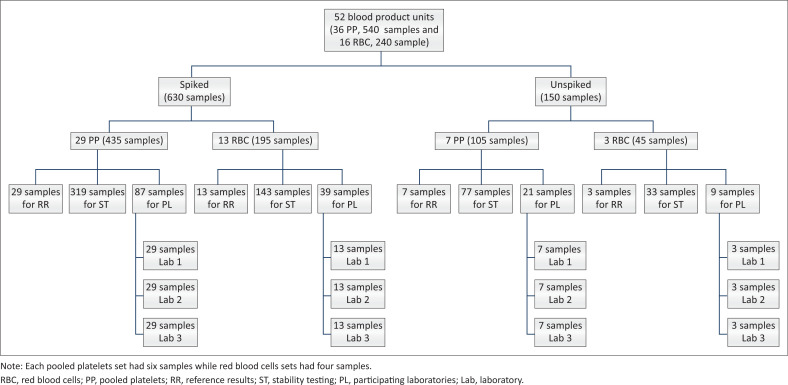
Sample split summary conducted at the South African National Blood Service in Johannesburg, South Africa, between April 2021 and June 2021.

Confirmation of intended results of manipulated units was performed after 48 h storage using Gram staining, bacterial culture, bacterial identification, and antimicrobial susceptibility testing (AST) by the researcher. This was followed by splitting of units; each unit was split into 15 samples (*n* = 780 PT samples in total). Samples were tested at three different stages, namely establishment of reference results (*n* = 52), stability testing (*n* = 572) and laboratory testing for evaluation purposes (*n* = 156). Sample identification is shown in Figure 1.

### Safety precautions and prevention of cross-contamination of samples during preparation

Aseptic technique was followed throughout the preparation of the PT matrix samples to prevent cross-contamination. The samples were prepared inside a Class II Biosafety cabinet (Esco Lifescience, Singapore) with one unit or bacterial strain at a time. The environment in which the preparation took place was monitored by taking samples from the air, surfaces and researchers’ hands.

### Testing of proficiency testing matrix samples

Testing of the prepared PT matrix was conducted in two stages, namely testing by the researcher and testing by the participating laboratories. All samples were subjected to bacterial culture and, if positive, Gram staining, bacterial identification and AST were performed. Two approved and commonly used bacterial culture testing platforms, where bacterial strains metabolise the substrates of the culture media, were used, namely BacT/ALERT 3D (bioMérieux, Craponne, France) and BACTEC 9120 (Becton Dickinson, San Jose, California, United States), and Vitek II Compact (bioMérieux, Craponne, France) was used for AST.

### Testing to establish reference results

Reference results of analysed spiked and non-spiked units had to confirm the absence and presence of bacterial growth and, if bacterial growth was observed, bacterial identification as well as AST pattern were determined. The reference results were expected to agree with the expected result per process. After confirmation of the reference results, the PT matrix samples were aliquoted into smaller 5 mL vials to prepare them for storage and independent testing by participating laboratories.

### Stability testing

Stability testing involved storing of samples at different temperature ranges (2 °C – 6 °C, 20 °C – 25 °C, and 37 °C) and random selection of these samples for testing on different days over a period of 30 days. A time period of 30 days was selected to ensure maximum time for analysis and stability testing. This time frame also allowed for sample transportation to participating laboratories, storage at participating laboratories prior to testing, analysis and submission of results to the researcher. The results obtained from all samples tested for stability on scheduled testing days were expected to be similar to the reference results.

### Testing by participating laboratories

Replicas of the same aliquoted samples were selected, three vials from each of the 52 units (*n* = 156). The 156 samples were further split into three sets of 52 identical samples which included spiked and unspiked samples. These samples were packed and transported at ambient temperature to three independent participating laboratories according to International Air Transport Association regulations.^15^ These laboratories are South African National Accreditation System accredited to International Organization for Standardization Standard 15189:2012. To ensure maintenance of temperature chain and validity of results, the participating laboratories were instructed to store the PT samples at ambient temperature until testing time. The samples were to be tested within 14 days of receipt according to their respective laboratory protocols. After testing, the participants sent the results to the researcher for evaluation against preset limits of acceptability and the reference results.

### Statistical analysis

Qualitative data obtained from the participating laboratories and from stability testing included two dependent variables, such as positive/negative, sensitive/resistant, correct/incorrect. Post capturing, qualitative data were coded and converted into a quantitative form, for example 1 for positive and 2 for negative, and analysed using Cohen’s kappa (*k*) statistical method. The Statistical Package for Social Sciences program (IBM, Bengaluru, India) was used to measure Cohen’s kappa. The inter-rater reliability agreement and the *p*-value cut-off points were calculated as: *k* > 0.75 and *p* ≤ 0.05, indicating statistical significance. The kappa score and *p*-value were evaluated between the researcher and the participating laboratories as well as for stability testing results.

## Results

### Reference and stability testing results

Reference testing results by the researcher were similar to results obtained during preparation of the matrices for spiked and the non-spiked units, thus 100% (52 out of 52) compliant results were obtained. Table 1 indicates the number of products spiked with a specific bacterial strain; for example, *S. epidermidis* was used to spike three PP and one RBC concentrates.

**TABLE 1:** Bacterial strains used to spike products at the South African National Blood Service in Johannesburg South Africa between April 2021 and June 2021.

Gram stain	Bacterial strain used for spiking†	Total (*n*) of PP concentrates inoculated	Total (*n*) of RBC concentrates inoculated
Gram-positive cocci (*n* = 14)	*Staphylococcus epidermidis*	3	1
	*Staphylococcus aureus*	2	1
	*Streptococcus pyogenes*	3	1
	*Streptococcus bovis*	3	1
	*Streptococcus dysgalacticae*	1	1
Gram-positive bacilli (*n* = 9)	*Bacillus cereus*	3	2
	*Bacillus thuringiensis*	1	1
	*Listeria monocytogenes*	1	1
Gram-negative bacilli (*n* = 19)	*Enterobacter cloacae*	3	1
	*Serratia marcescens*	2	1
	*Escherichia coli*	3	1
	*Morganella morganii*	3	1
	*Klebsiella pneumoniae*	1	1
	*Pseudomonas fluorescens*	1	1
	*Serratia liquefaciens*	1	1
No bacteria observed (*n* = 10)	Preserving fluid	7	3

**Total (*n* = 52)**	**-**	**36**	**16**

PP, pooled platelet; RBC, red blood cells.

† , The bacterial strains obtained from reference results were confirmed to be the same as the bacterial strains used for spiking of PP and RBC concentrates.

For stability testing, 52 out of 52 (100%) compliant results were obtained until day 14 for both spiked and unspiked units; thereafter, only PP and RBC units spiked with *L. monocytogenes* started showing signs of deterioration. From day 23, *M. morganii* failed to grow on PP and RBC units. A 99% (206 out of 208) level of agreement was achieved for stability testing from all variables tested.

### Bacterial culture and Gram stain level of agreement for stability testing

**FIGURE 2:**
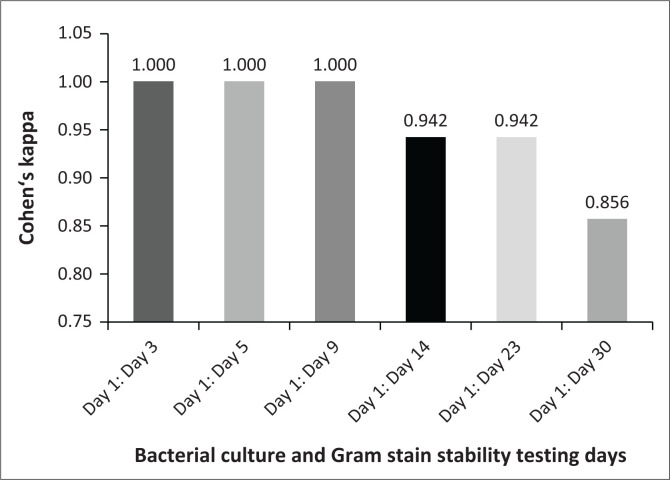
Kappa scores for bacterial culture and Gram stain stability testing for spiked and unspiked concentrates at the South African National Blood Service in Johannesburg, South Africa, between April 2021 and June 2021.

A high level of agreement was obtained across all testing days for bacterial culture results stability detection with kappa scores of > 0.75 (Figure 2). The *p*-value for all testing days was < 0.001. From day 1 until day 9, *k* = 1.000 was maintained. A decrease in the kappa score was observed from Day 9 to Day 14, where *k* = 0.942, and a further decline on Day 30, with *k* = 0.856.

### Bacterial identification level of agreement for stability testing

The stability testing for bacterial identification indicated a Cohen’s kappa score of 1.000 on Day 1 versus Day 3, Day 1 versus Day 5 and Day 1 versus Day 9, as illustrated in Table 2. Day 1 versus Day 14, as well as Day 1 versus Day 23, had a kappa score of 0.955, while Day 1 versus Day 30 indicated a score of 0.842. The *p*-value obtained across all the testing days was < 0.001.

**TABLE 2:** Bacterial identification stability testing kappa statistics for pairs of measures at the South African National Blood Service in Johannesburg, South Africa, between April 2021 and June 2021.

Stability testing days	Bacterial identification kappa scores	
	Kappa value	*p*
Day 1 versus Day 3	1.000	< 0.001
Day 1 versus Day 5	1.000	< 0.001
Day 1 versus Day 9	1.000	< 0.001
Day 1 versus Day 14	0.955	< 0.001
Day 1 versus Day 23	0.955	< 0.001
Day 1 versus Day 30	0.842	< 0.001

### Antimicrobial susceptibility testing level of agreement for stability testing

Gram-positive and Gram-negative results obtained from all AST cards utilised had a constant statistical value from Day 1 until Day 30. A high level of agreement for AST stability testing was obtained with *k* = 1.000, *p* < 0.001 across all testing days.

### Comparative results between the researcher (reference results) and participating laboratories

A high level of agreement (98% to 100%) between the reference standard and participating laboratories was obtained. Two percent of discrepant results were obtained for Gram stain and bacterial identification, while 98% of the results were comparable to the reference results. The results for all tested variables are summarised in Table 3.

**TABLE 3:** Antimicrobial susceptibility testing kappa statistics for pairs of measures between the researcher and the participating laboratories at the South African National Blood Service in Johannesburg, South Africa, between April 2021 and June 2021.

Microbial agents	AST kappa statistics for pairs of measures					
	Researcher: Lab 1	Researcher: Lab 2	Researcher: Lab 3	Lab 1: Lab 2	Lab 1: Lab 3	Lab 2: Lab 3
Bacterial culture	1.000	1.000	1.000	1.000	1.000	1.000
Gram stain	0.947	0.920	0.920	0.974	0.867	0.841
Bacterial identification	0.958	0.958	0.894	1.000	0.894	0.894
AST	1.000	1.000	1.000	1.000	1.000	1.000

AST, antimicrobial susceptibility testing; Lab, laboratory.

### Bacterial culture level of agreement between researcher and participating laboratories

Eighty-one percent (42 out of 52) of the samples were expected to yield positive bacterial culture results while 19% (10 out of 52) were expected to give negative results during testing by both participating laboratories and the researcher. A high level of agreement was obtained between all participating laboratories and the reference results, with *k* = 1.000 and *p* < 0.001 across all participating laboratories (Table 3).

### Gram stain level of agreement between researcher and participating laboratories

The samples for Gram stain consisted of 16 out of 52 (30.8%) Gram-positive cocci, 7 out of 52 (13.5%) Gram-positive bacilli, 19 out of 52 (36.5%) Gram-negative bacilli and 10 out of 52 (19.2%) with no bacteria observed. Laboratory 1 obtained a kappa score of 0.947, and Laboratory 2 and 3, 0.920, when compared to the researcher. Laboratory 2 and Laboratory 3 had kappa scores of 0.974 and 0.867 when compared to Laboratory 1, while Laboratory 3 had a 0.841 kappa score when compared to Laboratory 2. A high level of agreement, shown in Table 3, between the researcher and all participating laboratories was obtained as well as a *p*-value of < 0.001, across all participating laboratories.

### Bacterial identification level of agreement between researcher and participating laboratories

A high level of agreement, summarised in Table 3, was obtained across all participating laboratories and the researcher for bacterial identification. Laboratory 1 and Laboratory 2 had a 0.958 kappa score when compared to the researcher, while Laboratory 3 had a score of 0.894. Laboratory 3 had a kappa score of 0.894 when compared to Laboratory 1 and Laboratory 2. A kappa score of 1.000 was obtained between Laboratory 1 and 2. A statistically significant *p*-value of < 0.001 was obtained across all participating laboratories.

### Antimicrobial susceptibility testing level of agreement between researcher and participating laboratories

All participating laboratories obtained results similar to the researcher for AST, with *k* = 1.000, *p* < 0.001, indicating a high level of agreement and statistical significance across all participating laboratories, as illustrated in Table 3.

## Discussion

In this study, a stable microbiology PT programme using platelets and red blood cells as the basis matrix has been developed to be used on bacterial detection analysers. Unlike the commercially available lyophilised and frozen PT programmes, detection of active bacterial growth metabolism on blood products was observed in this study.^10^ There were no discrepancies observed between the reference results and the participating laboratories for bacterial culture and AST results. All positive and negative cases were identified correctly, showing a high level of agreement.

The deviations from the reference results observed do not discredit the newly developed PT matrices; however, non-complying results must be investigated. Participating laboratories must address non-complying results promptly according to appropriate quality management systems.^16^

The results obtained during stability testing demonstrated that storage time of up to 23 days did not influence the outcome of results for most bacterial strains, except for *L. monocytogenes*. The laboratory test results obtained during storage in different temperature ranges also confirmed that the newly developed PT matrices were stable.

Two out of 42 bacterially spiked samples started showing some degree of deterioration as they could not give a positive signal, from Days 14 and 23. The two non-compliant results were from both PP and RBC, and these were both spiked with *L. monocytogenes. Listeria monocytogenes* was unable to grow beyond Day 14 as it requires specific growth substances in addition to what was provided by the PP and RBC concentrates. These specific growth substances include amino acids such as leucine, methionine, arginine as well as vitamins such as riboflavin, thiamine, biotin and thioctic acid.^17^ In a study conducted by Prax et al., it is stated that *L. monocytogenes* was slow growing in cold stored red cells from Days 7 to 28.^18^ Both platelet and RBC bags were not supplemented with the required amino acids and vitamins to support the growth of *L. monocytogenes*. Brain Heart Infusion broth which has been confirmed to be effective in the cultivation of a wide range of microbes, could be considered as the base medium to augment growth. This broth contains protease peptone and other infusions which serve as sources of amino acids, vitamins as well as vital growth factors.^18^

At Day 30, acceptable agreements were obtained; however, 5 out of 42 bacterial spiked samples showed signs of deterioration for bacterial culture. Blood products spiked with *L. monocytogenes* and *M. morganii* were mostly affected. *Morganella morganii* could not be identified on Day 30 by either the researcher or the participating laboratories. This bacterial strain was validated and accepted during enlargement of the World Health Organization international repository for platelet transfusion.^19^ During that validation, *M. morganii* was spiked into platelet concentrates and stored for up to seven days at 20 °C – 25 °C; in comparison, the storage in this matrix-equivalent PT establishment study was for up to 30 days at 20 °C – 25 °C. Based on observation from this study, one can then conclude that *M. morganii* is stable for 23 days, as all PP and RBC concentrates spiked with this bacterial strain had growth until Day 23.

*Staphylococcus aureus* and *S. pyogenes* showed signs of deterioration at Day 30. One participating laboratory identified *Staphylococcus aureus* as *Staphylococcus sciuri* and *Streptococcus pyogenes* as *Alicyclobacillus acidoterrestris*, on Day 30. In a real case scenario, misidentification of bacteria could lead to patient mistreatment or incorrect treatment or no treatment at all; for example, when a Gram-positive coccus is identified instead of a Gram-negative bacillus, incorrect antimicrobial agents effective for killing or inhibiting only the growth of Gram-positive cocci may be used for treatment of patients. Bacterial identification discrepancies observed on Day 30 could have been caused by poor aseptic technique during testing on that day by the participating laboratory. The PP and RBC treated with preserving fluid remained negative throughout the study. It can therefore be concluded that no contamination occurred during storage of the PT matrices.

### Limitations

The study is currently limited to PT samples transported by road as stability testing was not performed on samples transported by air. The PT samples were only tested on bacterial culture methods detecting active bacterial metabolism and not on other platforms such as flow cytometry and rapid bacterial detection methods. Further findings indicated a limitation on *L. monocytogenes*’ inability to grow on platelet and RBC concentrates; this could be due to method limitation. It is recommended that growth of *L. monocytogenes* be enhanced by the addition of emulsified microbe into Brain Heart Infusion enrichment media prior to spiking of concentrates.^17^

### Conclusion

It was concluded that the majority of the newly developed blood PT matrices were stable for up to 23 days at all storage conditions between 2 °C and 37 °C; therefore, PT matrices will be suitable for sending to areas where delivery can be achieved within 7 days from dispatch. *Listeria monocytogenes* will be excluded from the PT matrices and further studies will be conducted.

This matrix will cover Gram staining, bacterial screening and identification as well as antimicrobial susceptibility testing. The blood product matrix will eliminate matrix-related problems such as biases and uncertainties caused by use of the inappropriate matrix (lyophilised and frozen) while performing PT. The outcome of this study, therefore, provided a possible solution for closing the gap of the unavailable blood PT matrix for transfusion microbiology. Further validation and comparison for reproducibility before being offered as an official PT programme for transfusion microbiology are suggested. Once implemented successfully locally, these PT samples will be validated for shipment to African countries and other countries beyond South African borders to support blood safety.
